# Overexpression of CTEN relates to tumor malignant potential and poor outcomes of adenocarcinoma of the esophagogastric junction

**DOI:** 10.18632/oncotarget.21109

**Published:** 2017-09-20

**Authors:** Kenichi Aratani, Shuhei Komatsu, Daisuke Ichikawa, Takuma Ohashi, Mahito Miyamae, Wataru Okajima, Taisuke Imamura, Jun Kiuchi, Keiji Nishibeppu, Toshiyuki Kosuga, Hirotaka Konishi, Atsushi Shiozaki, Hitoshi Fujiwara, Kazuma Okamoto, Hitoshi Tsuda, Eigo Otsuji

**Affiliations:** ^1^ Division of Digestive Surgery, Department of Surgery, Kyoto Prefectural University of Medicine, 465 Kajii-cho, Kawaramachihirokoji, Kamigyo-ku, Kyoto, Japan; ^2^ Department of Pathology, National Cancer Center Hospital, Tokyo, Japan; ^3^ Department of Basic Pathology, National Defense Medical College, Saitama, Japan

**Keywords:** adenocarcinoma of the esophagogastric junction, gastric cancer, CTEN, prognosis, oncogene

## Abstract

**Background:**

To detect a novel treatment target for adenocarcinoma of the esophagogastric junction (AEG), we tested whether C-terminal tensin-like (CTEN), a member of the tensin gene family and frequently overexpressed in various cancers, acts as a cancer-promoting gene through overexpression in AEG.

**Materials and Methods:**

We analyzed 5 gastric adenocarcinoma (GC) cell lines and 104 primary AEG tumors curatively resected in our hospital between 2000 and 2010.

**Results:**

CTEN overexpression was detected in GC cell lines (2/5 cell lines; 40%) and primary AEG tumor samples (35/104 cases; 34%). CTEN knockdown using several specific siRNAs inhibited the proliferation, migration, and invasion of CTEN-overexpressing cells. CTEN overexpression was significantly correlated with more aggressive venous and lymphatic invasion, deeper tumor depth, and higher rates of lymph node metastasis and recurrence. Patients with CTEN-overexpressing tumors had a worse overall rate of survival than those with non-expressing tumors (*P* < 0.0001, log-rank test) in an expression-dependent manner. CTEN positivity was independently associated with a worse outcome in the multivariate analysis (*P* = 0.0423, hazard ratio 3.54 [1.04–16.4]).

**Conclusions:**

CTEN plays a crucial role in tumor cell proliferation, migration, and invasion through its overexpression, which highlights its usefulness as a prognosticator and potential therapeutic target in AEG.

## INTRODUCTION

Over the past few decades, adenocarcinoma of the esophagogastric junction (AEG) has markedly increased in Western and Eastern countries [1–5]. Despite the improvement of diagnosis and treatment technologies over the past three decades such as extended radical resection and chemo and/or chemoradiotherapy, many AEG patients frequently develop metastasis and experience recurrence, and the long-term survival remains poor because of the aggressive and systemic nature of this disease [6].

Numerous genes have been analyzed to understand the molecular mechanisms of carcinogenesis and improve the clinical outcomes for adenocarcinoma of the esophagus and esophagogastric junction. Various genes with frequent alterations and molecular functions have been identified [7] such as amplification/overexpression of EGFR and ERBB2 [8]; hypermethylation or mutation of p16, APC, and TP53 [9, 10]; and overexpression/activation of c-Met and β-catenin [11]. However, in clinical settings, only a few genes have been used as diagnostic biomarkers and/or therapeutic targets [12]. We, therefore, wished to identify novel genes associated with the progression of AEG.

C-terminal tensin-like protein (CTEN, also known as Tensin4) is a novel focal adhesion protein belonging to the tensin family of proteins [13]. CTEN has been suggested to have oncogenic functions in various cancers [14–23]. Namely, CTEN, which promotes cell proliferation, migration, and invasion, is up-regulated by EGF [14], Stat3 [17], Kras signaling via Braf [18], FGF2 [22], and EGF-induced ERK1/2 [23]; interacts with β-catenin [15] and integrin-linked kinase (ILK) [20]; represses E-cadherin [16]; and modulates EGFR [21] and MET [24]. Although it showed tumor-suppressor functions in prostate cancer [13], in primary tumors, CTEN was significantly overexpressed in many types of primary cancers including thymoma, gastric, colorectal, breast, lung, skin, and pancreatic cancer [14, 15, 18–20, 25–32] and was a clinically relevant prognostic marker for these cancers. However, to date, there has been no report on the clinical and prognostic significance of CTEN in patients with primary AEG.

In this study, we tested whether CTEN acts as a cancer-promoting gene through its overexpression in AEG. Our results provided evidence that CTEN could be a target for molecular therapy and an important molecular marker for determining the malignant properties of tumors in patients with AEG.

## RESULTS

### Overexpression of CTEN in gastric cancer cell lines

**Figure 1:**
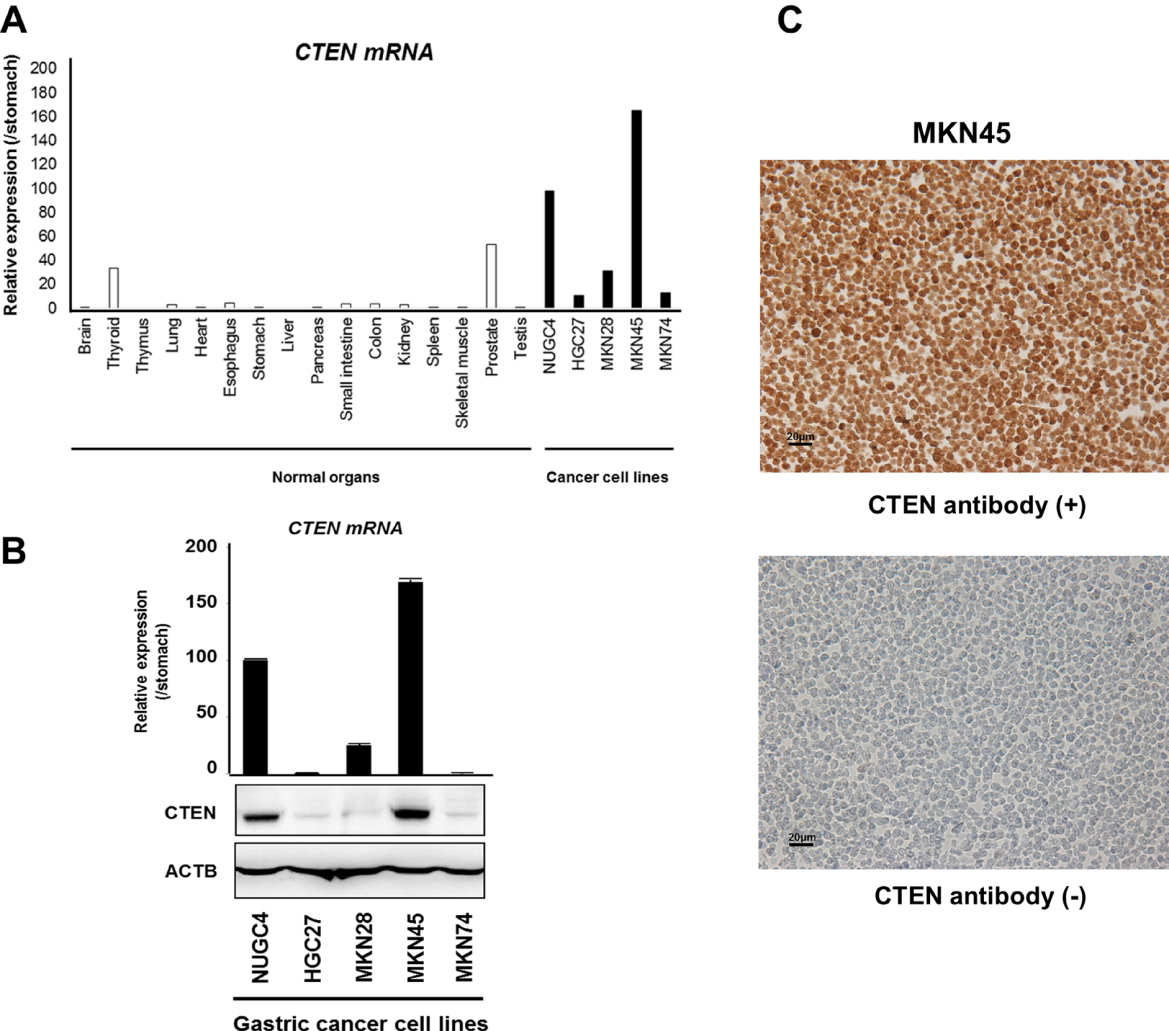
Expression profiles of CTEN in GC cell lines (**A**) Expression of CTEN mRNA in five GC cell lines compared with that in the normal organs. (**B**) Quantitative RT–PCR and western blotting analysis were performed using a CTEN-specific antibody to determine CTEN mRNA (top) and protein (bottom) expression in the gastric cancer cell lines NUGC4, HGC27, MKN28, MKN45, and MKN74. CTEN overexpression was observed in the NUGC4 and MKN45 cells (2/5 lines, 40%). (**C**) Formalin-fixed gastric cancer MKN45 cell line presenting the overexpression of CTEN, in which > 50% of cells showed staining, was used as a positive control, whereas a formalin-fixed gastric cancer HGC27 cell line (data not shown) and MKN45 staining without the CTEN antibody presenting low expression of CTEN was included as a negative control.

Quantitative RT-PCR analysis was performed to test whether CTEN was overexpressed in gastric cancer cell lines compared with the normal organs (Figure 1A). CTEN mRNA overexpression was observed in all gastric cancer cell lines NUGC4, HGC27, MKN28, MKN45, and MKN74. Western blotting analysis was performed using a CTEN-specific antibody to determine CTEN protein expression in the gastric cancer cell lines (Figure 1B). CTEN overexpression was observed in the NUGC4 and MKN45 cells (2/5 lines, 40%), suggesting that the CTEN gene was a target for activation in gastric cancer cell lines. Expression of CTEN protein correlated with that of CTEN mRNA in gastric cancer cell lines (Figure 1B). A formalin-fixed gastric cancer MKN45 cell line presenting the overexpression of CTEN, in which > 50% of cells showed staining, was used as a positive control, whereas a formalin-fixed gastric cancer HGC27 cell line (data not shown) and MKN45 staining without the CTEN antibody presenting low expression of CTEN was included as a negative control (Figure 1C).

### Immunohistochemical analysis of CTEN expression in primary AEG tumors

**Figure 2:**
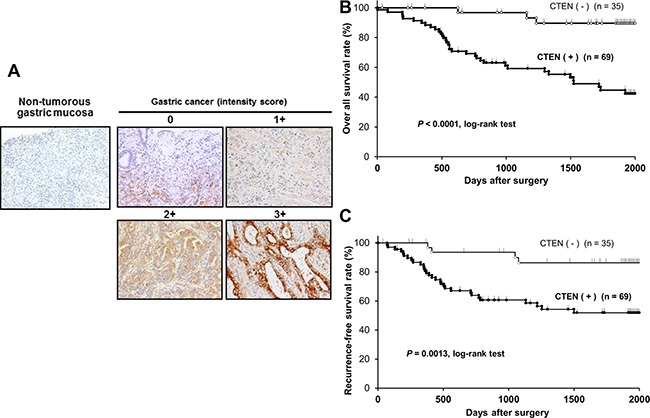
Immunohistochemical-staining analyses and postoperative overall and recurrence free survival curve according to the expression of CTEN (**A**) Specific immunostaining of the CTEN protein in primary samples was confirmed. Expression of the CTEN protein was observed in the cytoplasm of cancer cells. For the scoring of CTEN expression, the intensity score was defined as 0 = negative, 1 = weak, 2= moderate, or 3 = strong. (**B**) The CTEN high expression group had a significantly poorer prognosis than the low expression group in overall survival (*P* < 0.0001, log-rank test) (**C**) and recurrence free survival (*P* = 0.0013, log-rank test).

We examined the clinicopathological significance of CTEN expression in primary tumor samples of AEG based on the immunohistochemical staining pattern of this protein. CTEN expression was detected in the cytoplasm of AEG cells. We classified 104 AEG tumors into positive and negative groups according to the intensity of CTEN staining among tumor cells as described in MATERIALS AND METHODS. In primary cases, CTEN protein expression was negative in most of the non-tumorous esophagogastric mucosal cell population. We divided 104 AEG tumors into a high expression group with intensity score > 2 and ≥ 10% of tumor cells showing immunopositivity (*n* = 69, 66%) and a low expression group with intensity score ≤ 1 and/or < 10% of tumor cells showing immunopositivity (*n* = 35, 34%) according to the intensity of CTEN staining among tumour cells (Figure 2A). The high expression group had a significantly poorer prognosis than the low expression group for overall survival (*P* < 0.0001, log-rank test), (Figure 2B) and disease-free survival (*P* = 0.0013, log-rank test) (Figure 2C). The five-year overall survival rates of patients with CTEN high- and low-expression cancer in each stage were 75% vs. 100% (*P* = 0.023) in stage I, 34% vs. 68% (*P* = 0.074) in stage II or III, respectively.

Regarding the chemoresistant effect of CTEN in stage II and III patients, of all 67 patients with stage II and III cancer, 46 patients received adjuvant chemotherapy. Of these, the CTEN high-expression group (*n* = 37) had a poorer prognosis than the low-expression group (*n* = 9) for overall survival (5-year overall survival; CTEN high vs. low: 32% vs. 58%) and recurrence-free survival (5-year recurrence-free survival; CTEN high vs. low: 34% vs. 58%). Although there was no significant difference, CTEN overexpression may be associated with chemoresistant function. Concerning the recurrence patterns, of 104 patients, 34 patients had recurrences. As shown in Table 1, only 4 patients (11% (4/35)) with CTEN low-expression had a recurrence, and all of these were a peritoneal recurrence. In contrast, 31 patients (45% (31/69)) with CTEN high-expression had distant lymph node (*n* = 11), peritoneal (*n* = 6), liver (*n* = 4), or other recurrences (*n* = 10). There was no significant difference between both groups.

**Table 1:** Association between clinicopathological characteristics and CTEN expression

		*n*	CTEN immunoreactivity				*P*-value*
**high expression**		**low expression**	
Total		104	69		35		
Sex	male	82	55	(80%)	27	(77%)	
	female	22	14	(20%)	8	(23%)	0.8022
Age (y)	mean	66 (range:28−85)					
	< 65	45	26	(38%)	19	(54%)	
	> 65	59	43	(62%)	16	(46%)	0.1428
Siewert classification	I	12	7	(10%)	5	(14%)	
	II	60	42	(61%)	18	(51%)	
	III	32	20	(29%)	12	(34%)	0.6351
Histopathological grading	differe.	57	40	(58%)	17	(49%)	
	undiffere.	47	29	(42%)	18	(51%)	0.4083
Tumour size (mm)	< 40	39	21	(30%)	18	(51%)	
	> 40	65	48	(70%)	17	(49%)	0.0532
Venous invasion	0	49	24	(35%)	25	(71%)	
	1	29	22	(32%)	7	(20%)	
	2	13	10	(14%)	3	(9%)	
	3	13	13	(19%)	0	(0%)	**0.0018**
Lymphatic invasion	0	41	21	(30%)	20	(57%)	
	1	32	21	(30%)	11	(31%)	
	2	11	9	(13%)	2	(6%)	
	3	20	18	(26%)	2	(6%)	**0.0155**
TNM classification							
pT categories	T1	31	11	(16%)	20	(57%)	
	T2	15	9	(13%)	6	(17%)	
	T3	29	25	(36%)	4	(11%)	
	T4	29	24	(35%)	5	(14%)	**< 0.0001**
pN categories	N0	55	29	(42%)	26	(74%)	
	N1	9	5	(7%)	4	(11%)	
	N2	16	11	(16%)	5	(14%)	**0.0006**
	N3	24	24	(35%)	0	(0%)	
pStage	I	37	15	(22%)	22	(63%)	
	II	17	11	(16%)	6	(17%)	
	III	50	43	(62%)	7	(20%)	**< 0.0001**
Recurrence	absent	69	38	(55%)	31	(89%)	
	present	35	31	(45%)	4	(11%)	**0.0008**

NOTE. Statistically significant values are in bold type.

*P-values are from χ^2^ or Fisher's exact test and were statistically significant at < 0.05.

### Association between CTEN protein levels and clinicopathological characteristics in primary AEG

The relationship between the expression of the CTEN protein and clinicopathological characteristics is summarized in Table 1. The protein expression of CTEN was significantly associated with more aggressive venous (*P* = 0.0018) and lymphatic invasion (*P* = 0.0155), deeper depth of invasion (*P* < 0.0001), and higher rates of lymph node metastasis (*P* = 0.0006) and recurrence (*P* = 0.0008), whereas other characteristics including the Siewert classification and histological grade were not. In the Cox proportional hazard regression model (Table 2), univariate analyses demonstrated that CTEN protein expression, venous invasion, lymphatic invasion, pT category, and pN category were significantly associated with overall survival. When the data were stratified for multivariate analysis using the Cox proportional hazards analysis procedures, CTEN immunoreactivity in tumor cells remained significant (*P* = 0.0423, hazard ratio, 3.5 [1.04–16.4]) for overall survival in all patients, suggesting that CTEN immunoreactivity can be an independent predictor of overall survival.

**Table 2:** Multivariate analysis using the stepwise Cox regression procedures

Variables	Univariate^a^		Multivariate^b^			
***P*-value**	**HR^c^**	**95% CI^d^**			***P*-value**
Sex						
male *versus* female	0.8606			−		
Age						
> 65 *versus* < 65	0.5975			−		
Siewert classification						
l *versus* ll-lll	0.2602			−		
Histological type						
undiffe. *versus* diffe.	0.0816			−		
Tumour size (mm)						
> 40 *versus* < 40	0.0502			−		
Venous invasion						
positive *versus* negative	**< 0.0001**			−		
Lymphatic invasion						
positive *versus* negative	**< 0.0001**			−		
pT-stage						
T4 *versus* T1-3	**< 0.0001**	3.184	1.570	−	6.747	**0.0012**
pN-stage						
N2-3 *versus* N0-1	**< 0.0001**	3.149	1.423	−	7.624	**0.0040**
CTEN expression						
high *versus* low	**< 0.0001**	3.989	1.393	−	16.84	**0.0073**

^a^Kaplan and Meier method, for which the statistical significance was determined by log-rank test.

^b^Multivariate survival analysis was performed using Cox's proportional hazard model.

^c^HR: hazard ratio.

^d^CI: confidence interval

### Suppression of cell proliferation by downregulation of CTEN expression

**Figure 3:**
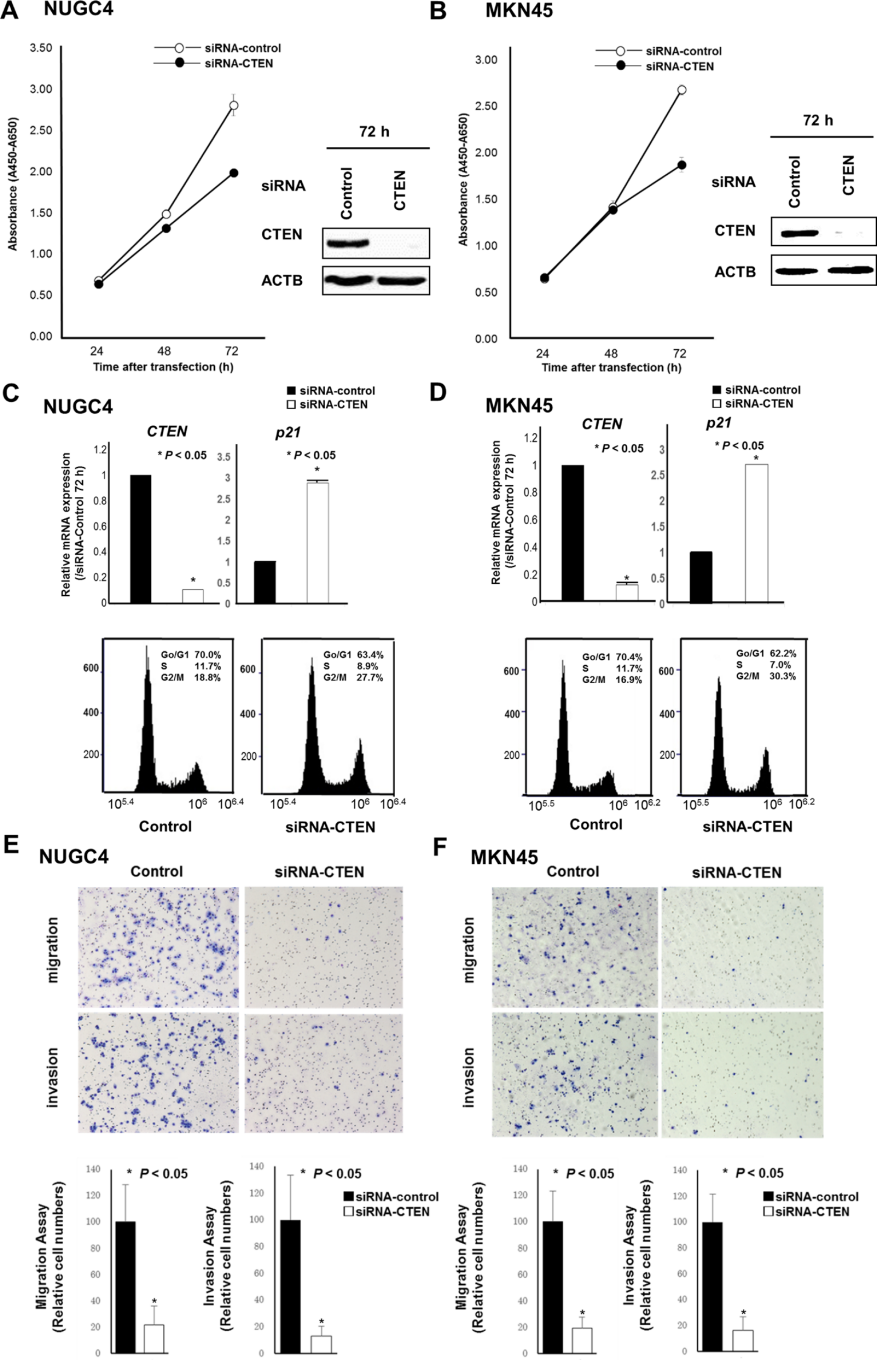
Effects of downregulation of CTEN expression Loss-of-function screening was undertaken using small interfering RNA (siRNA) targeting CTEN in the NUGC4 and MKN45 cells. To assess the cell growth, the numbers of viable cells at various time points after transfection were assessed by the colorimetric water-soluble tetrazolium salt (WST) assay. The knockdown of a target CTEN gene was confirmed by western blotting analysis (**A**, **B**). In both cell lines, p21 abundance was increased at 72 h by the knockdown of CTEN, which was confirmed by quantitative RT-PCR. FACS analysis of NUGC4 and MKN45 cells with transfection by siRNA-CTEN compared with control siRNA (**C**, **D**). Transwell migration and invasion assays using siRNA targeting CTEN (**E**, **F**).

To gain insight into the potential role of CTEN as an oncogene whose overexpression could be associated with AEG carcinogenesis, we first performed a cell proliferation assay using siRNAs specific to CTEN to investigate whether knockdown of CTEN expression could suppress the proliferation of gastric cancer cells showing overexpression of the gene. In NUGC4 and MKN45 cell lines, expression of the CTEN protein and mRNA was more efficiently knocked down 24–72 h after the transient introduction of a CTEN-specific siRNAs (siRNA-CTEN) than with the control siRNA (siRNA-control) (72 h knockdown effect in CTEN protein expression; Figure 3A and 3B). The proliferation of NUGC4 and MKN45 cells were 29.3% and 30.1% lower than with controls after the knockdown of endogenous CTEN expression.

### Cell cycle analysis by downregulation of CTEN expression using fluorescence-activated cell sorting (FACS)

Fluorescence activated cell sorting (FACS) analysis demonstrated that transfection of NUGC4 and MKN45 cells with siRNA-CTEN resulted in an accumulation of cells in the G2/M phase compared with transfection with control siRNA (Figure 3C and 3D). In addition, in both cells, p21 abundance was increased at 72 h mRNA level in CTEN transfected cells (Figure 3C and 3D). These findings suggested that the knockdown of CTEN overexpression in these cells induced the production of p21, which resulted mainly in G2/M arrest.

### Suppression of cell migration and invasion by knockdown of CTEN

Transwell migration and invasion assays were performed to examine the invasive potential of both NUGC4 and MKN45 cells transfected with siRNA-CTEN to move through pores under different conditions. An uncoated membrane was used for the migration assays, whereas a Matrigel-coated membrane was used for the invasion assays. The number of both NUGC4 and MKN45 cells that migrated into the lower chamber was significantly lower for siRNA-CTEN-transfected cells than for siRNA-control-transfected cells under both conditions (Figure 3E and 3F), suggesting that CTEN may increase the ability of gastric cancer cells to migrate and invade.

## DISCUSSION

CTEN (C-terminal tensin-like, aka tensin4, TNS4) gene is the forth member of the tensin family (tensin-1, -2, -3 and -4 (cten)), maps to chromosome 17q12-q21, and encodes a 715-amino acid protein. It was identified as a distant member of the tensin focal adhesion family [13]. It is a much smaller protein compared to the other tensins and shares only the SH2 (Src homology 2) and PTB (phosphotyrosine binding) domains found at the C-terminal ends of all other tensins [33]. CTEN was originally identified with tissue-specific expression in the prostate and placenta [13]. Although it may not be expressed in other normal tissues, CTEN expression has been found to increase significantly in many types of cancer including thymoma, gastric, colorectal, breast, lung, skin, pancreatic, and hepatocellular cancer [14, 15, 18–20, 25–32], suggesting that overexpression of CTEN in these organ tissues may play a critical role in tumorigenesis. However, CTEN expression has not yet been documented in AEG.

In this study, we hypothesized that the overexpression/activation of CTEN may contribute to malignant tumor behavior and poor outcomes in AEG and gastric cancer. To verify this hypothesis, we examined the expression status of CTEN and its relation to clinicopathological factors as well as the biological significance of its expression in primary tumor tissues of AEG and gastric cancer cell lines. As a result, we demonstrated that CTEN was overexpressed in 48% (29/60) of primary AEG tissues and 57% (4/7) of cell lines, and CTEN overexpression was significantly correlated with more aggressive venous and lymphatic invasion, deeper tumor depth, and higher rates of lymph node metastasis and recurrence. Moreover, CTEN overexpression was a poor prognosticator independent of other prognostic factors and had the potential of resistance to chemotherapy in this study, although there was no significance. In addition, downregulation of CTEN expression suppressed cell proliferation, migration, and invasion in gastric cancer cells. These results suggested that CTEN could be a target for molecular therapy and an important molecular marker for predicting the malignant properties of tumours in patients with AEG.

Regarding previous studies on CTEN, Sakashita et al. showed that TNS4 (CTEN) was frequently overexpressed in primary gastric cancer tissues, and gastric cancer patients with high TNS4 (CTEN) mRNA expression significantly had a poor prognosis associated with lymph node metastasis and peritoneal dissemination. However, in their study, they did not clarify the molecular mechanisms of CTEN through cell proliferation, migration, and invasion assays in gastric cancer cells [30]. Hung et al. showed that overexpression of CTEN promoted cell migration, and FGF2 treatment failed to further enhance cell migration. However, their study was performed in colon cancer cells (SW480) and prostate epithelial cells (MLC-SV40) [22]. In this study, we clearly demonstrated the clinical behavior and molecular mechanisms of CTEN in AEG tissue and gastric cancer cells. Therefore, we suggest that our results are novel and important for understanding the molecular functions of CTEN in AEG and gastric cancer.

A previous study has identified that CTEN expression correlated with high EGFR and HER2 expression in invasive breast cancer [14]. This study also demonstrated that treatment of inflammatory breast cancer patients with an EGFR/HER2 dual-specificity kinase inhibitor significantly downregulated CTEN expression [14]. Because it is a monoclonal antibody against HER2, trastuzumab in combination with chemotherapy (ToGa study) can also be considered as a new standard option for patients with HER2-positive advanced gastric cancer and AEG [12], and this treatment contributes to survival prolongation of AEG. Although there has been no report that trastuzumab downregulates CTEN expression, the issue is currently under evaluation, and we will report the effect of trastuzumab on CTEN in the near future. Anyway, the incidence of HER2 overexpressing esophagogastric tumors is reported as only 15–30% [12]. Therefore, novel biomarkers and targeted agents are urgently needed. CTEN also might be a key molecule for selecting prospective patients with malignant outcomes associated with HER2 expression in AEG patients undergoing this chemotherapy. This issue is under evaluation.

In conclusion, this is the first report demonstrating that CTEN has a pivotal oncogenic role and is an independent, poor prognostic factor in AEG. Until now, there has been no report concerning an inhibitor of CTEN. However, such an inhibitor may be a potential therapeutic targeting agent in AEG. Also, studies of larger cohorts are needed to validate these findings before moving to a clinical setting. Our results suggest that CTEN plays a crucial role in tumor cell proliferation, migration, and invasion through its overexpression, which highlight its usefulness as a prognosticator and potential therapeutic target in AEG.

## MATERIALS AND METHODS

### Primary AEG tissue samples and gastric cancer cell lines

Primary tumor samples of AEG were obtained from 104 consecutive AEG patients, who had undergone curative resection at the Division of Digestive Surgery, Department of Surgery, Kyoto Prefectural University of Medicine (Kyoto, Japan) between 2000 and 2010. Samples were embedded in paraffin after 24 h of formalin fixation. Relevant clinical and survival data were available for all patients. Written consent was always obtained in the formal style and after approval by the local ethics committee. None of these patients underwent endoscopic mucosal resection, palliative resection, preoperative chemotherapy, or radiotherapy, and none of them had synchronous or metachronous multiple cancers in other organs. Two patients with stage I cancer underwent adjuvant chemotherapy, whereas 46 of 67 stage II and III patients received adjuvant chemotherapy. None of these patients received radiotherapy after surgery. Disease stage was defined in accordance with the International Union Against Cancer Tumor-Lymph Node-Metastases (TNM) classification (7th Edition) [34]. The mean follow-up period for surviving patients was 51.2 months.

In Japanese and Asian patients with AEG defined by the Siewert classification [35], most AEG tumors were classified as Siewert type II or III, which are also categorized as gastric cancer [3, 4]. Indeed, at our institute, 88% (92/104) of AEG tumors were classified as Siewert type II or III tumors. Therefore, we used gastric cancer cell lines to elucidate the molecular function of CTEN. Five gastric cancer cell lines, NUGC4, HGC27, MKN28, MKN45, and MKN74, were used in this study. HGC27 cells were cultured in Dulbecco's Minimum Essential Medium (DMEM): F12 medium, and the other cells were cultured in Roswell Park Memorial Institute (RPMI)-1640 medium (Sigma, St. Louis, MO). All media were purchased from Sigma and supplemented with 100 mL/L FBS (Trace Scientific, Melbourne, Australia). All cell lines were cultured in 50 mL/L carbon dioxide at 37°C in a humidified chamber.

### Quantitative RT–PCR

Single-stranded complementary DNAs generated from total RNA were amplified with primers specific for each gene. Levels of messenger RNA (mRNA) expression were measured by quantitative real-time fluorescence detection (ABI StepOnePlus^TM^ Sequence Detection System; Applied Biosystems, Foster City, CA) using TaqMan Gene Expression Assays (Hs00262662_m1 for CTEN and Hs00355782_m1 for p21; Applied Biosystems) according to the manufacturer's instructions. The total RNA of normal organs was purchased from Takara Bio Inc., Shiga, JAPAN (Human Total RNA Master Panel II (Cat No. 636643) and Human Liver Total RNA (Cat No. 636531)) and BioChain Institute Inc., CA, USA (Human Esophagus Total RNA (Cat No. R1234106-50)). The results of gene expression were calculated as the ratio between CTEN or p21 and an internal reference gene (Hs99999903_m1 for β-actin; Applied Biosystems) that provides a normalisation factor for the amount of RNA isolated from a specimen. This assay was performed in duplicate for each sample.

### Western blotting

Anti-CTEN mouse monoclonal antibody (clone 684524) was purchased from R&D Systems (Minneapolis, MN), and anti-ACTB antibody was purchased from Santa Cruz Biotechnology (Santa Cruz, CA). CTEN is an affinity purified mouse polyclonal antibody raised against a recombinant protein of CTEN. The cells were lysed, and their proteins were extracted using the M-PER^®^ Mammalian Protein Extraction Reagent (Thermo Scientific, USA).

### Loss-of-function by small interfering RNA (siRNA) and cell growth analysis

For the knocking down of endogenous CTEN expression, each of the small interfering RNAs (siRNA) targeting CTEN (Silencer^®^ Select siRNAs, siRNA-TNS4-1; # s39736; siRNA-TNS4-2; # s39737; siRNA-TNS4-3; # s39738; ThermoFisher, Waltham, Massachusetts, US) and the control were transfected into cells (6 nmol/L) using Lipofectamine RNAiMAX (Invitrogen, Carlsbad, CA) according to the manufacturer's instructions. The knockdown of a target gene was confirmed by quantitative RT-PCR and western blotting. To measure cell proliferation, the number of viable cells at 24, 48, and 72 h after siRNA transfection was assessed by the colourimetric water-soluble tetrazolium salt (WST) assay (Cell Counting Kit-8; Dojindo Laboratories, Kumamoto, Japan). Cell cycle stage was evaluated 72 h after transfection by fluorescence-activated cell sorting (FACS) as described elsewhere [36].

### Transwell migration and invasion assays

Transwell migration and invasion assays were carried out in 24-well modified Boyden chambers (transwell-chamber, BD Transduction, Franklin Lakes, NJ). The upper surface of the 6.4 mm diameter filters with 8-μm pores was pre-coated with (invasion assay) or without (migration assay) Matrigel (BD Transduction). The siRNA transfectants (2 × 10^4^ cells per well) were transferred into the upper chamber. Following 48 h of incubation, migrated or invasive cells on the lower surface of filters were fixed and stained with the Diff-Quik stain (Sysmex, Kobe, Japan), and stained cell nuclei were counted directly in triplicate. We assessed the invasive potential by calculating the ratio of the percentage invasion through the Matrigel-coated filters relative to the migration through the uncoated filters of test cells over that in the control counterparts, as described elsewhere [37–40].

### Immunohistochemistry

Anti-CTEN monoclonal antibody (clone 684524), which was the same antibody used in the western blotting analysis and was purchased from R&D Systems (Minneapolis, MN), was used. Primary tumor samples were fixed with 10% formaldehyde in PBS and routinely embedded in paraffin. The horseradish peroxidase (HRP) staining method was used. In brief, after deparaffinization, antigen retrieval was performed by heating the samples in 10 mmol/L citrate buffer (pH 6.0) at 95°C for 60 min. Endogenous peroxidases were quenched by incubating the sections for 20 min in 3% H_2_O_2_. After treatment with Block Ace (Dainippon Sumitomo Pharmaceutical, Osaka, Japan) for 30 min at room temperature, sections were incubated at room temperature for 1 hour with anti-CTEN (1:500). PBS was used for all dilutions and washings. The bound primary antibody was detected with EnVision™+ Horse Radish Peroxidase Systems (EnVision + dual link System-HRP; Dako North America, Inc., Carpinteria, CA). HRP labeling was visualized using color development with diaminobenzidine tetrahydrochloride. Slides were counterstained with Mayer's haematoxylin. A formalin-fixed gastric cancer cell line overexpressing CTEN (MKN45 cells), in which > 50% of cells showed staining of PBK/TOPK protein, was used as a positive control, whereas MKN45 cells incubated without the PBK/TOPK antibody were used as a negative control (Figure 1B).

To evaluate CTEN expression, in the percentage of tumor cells showing CTEN immunopositivity, primary tumors with at least 10% or more of the total cell population was judged positive, and less than 10% was judged negative. For the intensity of CTEN expression, the intensity score (0 = negative, 1 = weak, 2 = moderate, or 3 = strong) was examined. Namely, primary tumors with non-detectable CTEN expression, which was similar to non-tumorous gastric mucosa and stroma, were given an intensity score of 0, whereas those with the greatest CTEN abundance were given an intensity score of 3. The remaining tumors were categorized with intensity scores of 1 or 2 according to the intensity of immunohistochemical staining for CTEN. The expression of CTEN was regarded as high expression for both an intensity score > 2 and ≥ 10% of tumor cells showing immunopositivity or low expression for an intensity score ≤ 1 and/or < 10% using high-powered (×200) microscopy [40, 41].

### Statistical analysis

Clinicopathological variables pertaining to the corresponding patients were analyzed for statistical significance using the chi-squared test or Fisher's exact test. For the analysis of survival, Kaplan–Meier survival curves were constructed for groups based on univariate predictors, and differences between the groups were analyzed with the log-rank test. Univariate and multivariate survival analyses were performed using the likelihood ratio test of the stratified Cox proportional hazards model. Differences between subgroups were tested with the non-parametric Mann–Whitney *U*-test. Differences were assessed with a two-sided test and were considered statistically significant at *P* < 0.05.
