# Correction: Overexpression of CTEN relates to tumor malignant potential and poor outcomes of adenocarcinoma of the esophagogastric junction

**DOI:** 10.18632/oncotarget.27229

**Published:** 2019-10-01

**Authors:** Kenichi Aratani, Shuhei Komatsu, Daisuke Ichikawa, Takuma Ohashi, Mahito Miyamae, Wataru Okajima, Taisuke Imamura, Jun Kiuchi, Keiji Nishibeppu, Toshiyuki Kosuga, Hirotaka Konishi, Atsushi Shiozaki, Hitoshi Fujiwara, Kazuma Okamoto, Hitoshi Tsuda, Eigo Otsuji

**Affiliations:** ^1^ Division of Digestive Surgery, Department of Surgery, Kyoto Prefectural University of Medicine, 465 Kajii-cho, Kawaramachihirokoji, Kamigyo-ku, Kyoto, Japan; ^2^ Department of Pathology, National Cancer Center Hospital, Tokyo, Japan; ^3^ Department of Basic Pathology, National Defense Medical College, Saitama, Japan

**This article has been corrected:** A line in the Abstract section has been corrected to read, “CTEN overexpression was detected in GC cell lines (2/5 cell lines; 40%) and primary AEG tumor samples (69/104 cases; 66%).” The data in the main document and Table 1, Figure 2A remains correct. The authors declare that these corrections do not change the results or conclusions of this paper.

## ABSTRACT

**Background:** To detect a novel treatment target for adenocarcinoma of the esophagogastric junction (AEG), we tested whether C-terminal tensin-like (CTEN), a member of the tensin gene family and frequently overexpressed in various cancers, acts as a cancer-promoting gene through overexpression in AEG.

**Materials and Methods:** We analyzed 5 gastric adenocarcinoma (GC) cell lines and 104 primary AEG tumors curatively resected in our hospital between 2000 and 2010.

**Results:** CTEN overexpression was detected in GC cell lines (2/5 cell lines; 40%) and primary AEG tumor samples (69/104 cases; 66%). CTEN knockdown using several specific siRNAs inhibited the proliferation, migration, and invasion of CTEN-overexpressing cells. CTEN overexpression was significantly correlated with more aggressive venous and lymphatic invasion, deeper tumor depth, and higher rates of lymph node metastasis and recurrence. Patients with CTEN-overexpressing tumors had a worse overall rate of survival than those with non-expressing tumors (*P* < 0.0001, log-rank test) in an expression-dependent manner. CTEN positivity was independently associated with a worse outcome in the multivariate analysis (*P* = 0.0423, hazard ratio 3.54 [1.04–16.4]).

**Conclusions:** CTEN plays a crucial role in tumor cell proliferation, migration, and invasion through its overexpression, which highlights its usefulness as a prognosticator and potential therapeutic target in AEG.

Original article: Oncotarget. 2017; 8:84112–84122. 84112-84122. https://doi.org/10.18632/oncotarget.21109

